# tRNA-derived small RNA, tsRNA-5017b, as a novel biomarker for predicting severity in severe fever with thrombocytopenia syndrome

**DOI:** 10.1128/spectrum.00033-26

**Published:** 2026-05-26

**Authors:** Wanying Zhang, Jiawei Feng, Shixing Chen, Na Cao, Shuo Ma, Jiale Li, Linyan Gong, Haiyan Gao, Tianyang Liu, Xiaofeng Cheng, Hongpan Xu, Yuxin Chen

**Affiliations:** 1 Department of Laboratory Medicine, Nanjing Drum Tower Hospital Clinical College of Nanjing Medical University 12461, Nanjing, Jiangsu, China; 2 Department of Infectious Diseases, Nanjing Drum Tower Hospital, Affiliated Hospital of Medical School, Nanjing University 12581, Nanjing, China; 3 Department of Clinical Laboratory, Nanjing Central Hospital 687427 , Nanjing, China; 4 Laboratory Medicine, Zhongda Hospital, Medical School of Southeast University 66334, Nanjing, Jiangsu, China; 5 Department of Clinical Laboratory Medicine, Nanjing Drum Tower Hospital, Clinical College, Jiangsu University 12676, Zhenjiang, Jiangsu, China; 6 Department of Infectious Diseases, Nanjing Drum Tower Hospital Clinical College of Nanjing University of Chinese Medicine 66506, Nanjing, Jiangsu, China; 7 Department of Laboratory Medicine, Joint Institute of Nanjing Drum Tower Hospital for Life and Health, College of Life Science, Nanjing Normal University 12534, Nanjing, Jiangsu, China; 8 Department of Cardio-thoracic Surgery, Nanjing Drum Tower Hospital Clinical College of Traditional Chinese and Western Medicine, Nanjing University of Chinese Medicine 66478, Nanjing, China; 9 State Key Laboratory for Diagnosis and Treatment of Severe Zoonotic Infectious Diseases 543681 , Wuhan, Hubei, China; Fudan University, Shanghai, China

**Keywords:** severe fever with thrombocytopenia syndrome, SFTSV, tRNA-derived small RNA, biomarker

## Abstract

tRNA-derived small RNAs (tsRNAs) represent a novel class of highly conserved non-coding RNAs that play crucial roles in various cellular processes, including cell proliferation and the initiation of viral reverse transcription. Despite their significant biological functions, studies exploring the role of tsRNAs in infectious diseases remain limited. Severe fever with thrombocytopenia syndrome (SFTS) is an emerging tick-borne hemorrhagic fever caused by SFTS virus (SFTSV), which is associated with a high fatality rate. This study investigates the potential diagnostic value of serum tsRNAs in SFTS. Using tsRNA sequencing, we analyzed the differential expression of serum tsRNAs between SFTS patients and healthy controls, followed by validation using quantitative real-time PCR (qRT-PCR) in individual samples. Our findings revealed that the expression levels of tsRNA-3002a, tsRNA-5007a, tsRNA-5013c, tsRNA-5017b, tsRNA-5027b, and tsRNA-5030c were significantly altered in the serum of SFTS patients compared to healthy controls. Among these, tsRNA-5017b showed the most pronounced upregulation, with its expression progressively increasing in parallel with the severity of the disease. Further analysis demonstrated that tsRNA-5017b alone could predict SFTS severity at admission with a specificity of 93%. When combined with conventional laboratory markers such as platelet count (PLT) and aspartate aminotransferase (AST), the sensitivity reached 91.7%. Bioinformatics analysis suggested that tsRNA-5017b may be involved in the regulation of immune responses, apoptosis, and inflammatory pathways. In conclusion, this study is the first to identify the expression of tsRNA in the serum of SFTS patients and proposes tsRNA-5017b as a promising biomarker for predicting disease severity at admission.

**IMPORTANCE:**

Severe fever with thrombocytopenia syndrome (SFTS) is a fatal tick-borne disease caused by severe fever with thrombocytopenia syndrome virus (SFTSV), with limited early diagnostic tools available. This study identifies serum tRNA-derived small RNAs (tsRNAs) as potential biomarkers for SFTS, revealing that tsRNA-5017b, in particular, correlates with disease severity. Our findings show that tsRNA-5017b is associated with SFTS severity at admission, with high specificity alone and improved sensitivity when combined with conventional markers. This research highlights the potential of tsRNA-based biomarkers in diagnosing and assessing disease severity in SFTS, advancing our understanding of viral pathogenesis and immune regulation in infectious diseases.

## INTRODUCTION

tRNA-derived small RNAs (tsRNAs) are a class of non-coding RNAs produced by the specific cleavage of mature or precursor tRNAs by nucleases such as Dicer or angiogenin (ANG), typically under stress conditions or in specific tissues (1). These small RNAs are highly abundant, second only to microRNAs (miRNAs). tsRNAs are primarily generated by the precise processing of the 5′ or 3′ ends of mature tRNA or pre-tRNA, which can be classified into two subtypes: tRNA fragments (tRFs) and tRNA-derived initiator RNAs (tiRNAs) (2). They play crucial roles in various biological processes, including cell proliferation, initiation of viral reverse transcription, regulation of gene expression, RNA modification, DNA damage response, neurodegeneration, and tumor progression (3). In addition, a recent study has shown that alterations in premature tRNA expression and the formation of tRNA fragments are observed during viral infections, such as gammaherpesvirus, highlighting the dynamic involvement of tRNA-related small RNAs in host-pathogen interactions (4). tsRNAs are evolutionarily conserved from bacteria to humans (5), exhibiting organ-specific abundance. This conservation provides broad applicability and minimizes the potential interference from species- or individual-specific variations. As part of liquid biopsy, tsRNAs can be detected in various biological fluids, such as plasma or serum, offering a non-invasive and easily repeatable sampling method, making them ideal for high-throughput and automated processing. Although tsRNAs and miRNAs are similar in size, studies have shown that their expression profiles differ significantly in body fluids (6). For example, tRF-3001a and tRF-1003 have been shown to be superior biomarkers compared to their corresponding miRNAs (7). Furthermore, elevated levels of tsRNAs have been found in nasopharyngeal swab samples from patients infected with severe acute respiratory syndrome coronavirus 2 (SARS-CoV-2), as well as in those infected with respiratory syncytial virus (RSV), hepatitis B virus (HBV), and hepatitis C virus (HCV) (8). Given these advantages, tsRNAs hold great potential as molecular biomarkers in infectious diseases.

Severe fever with thrombocytopenia syndrome (SFTS) is an emerging tick-borne hemorrhagic fever caused by SFTS virus (SFTSV). Clinically, SFTS is characterized by fever, thrombocytopenia, leukopenia, and multi-organ dysfunction. In severe cases, patients may succumb to complications such as hemorrhage, disseminated intravascular coagulation (DIC), or secondary infections (9). Since its identification in China in 2011, SFTS has spread to other East Asian countries, including South Korea and Japan. The case fatality rate ranges from 12% to 30%, posing a serious public health threat (10). Current clinical prediction models for assessing the severity of SFTSV infection rely mainly on indicators such as age, activated partial thromboplastin time (APTT), d-dimer, prothrombin time (PT), and neurological manifestations (11–13). However, these models primarily depend on clinical features, laboratory indicators, or plasma proteins, without considering virus-specific products. This highlights the urgent need for a more effective prediction model. A comprehensive analysis of tsRNA profiles in SFTS patients, and their correlation with clinical parameters, could provide new insights into disease pathogenesis and help identify potential biomarkers for disease monitoring. However, studies on the expression profiles and clinical significance of tsRNAs in the serum of SFTS patients are still limited.

In this study, we aim to explore whether a distinct tsRNA profile could serve as a molecular fingerprint for SFTS. We identified differentially expressed tsRNAs in the serum of SFTS patients using tsRNA sequencing, followed by validation using quantitative real-time PCR (qRT-PCR). Additionally, we integrated clinical data to explore the association between altered tsRNA levels and disease severity, as well as organ injury markers such as alanine aminotransferase (ALT), aspartate aminotransferase (AST), and lactate dehydrogenase (LDH). The candidate tsRNAs were clinically evaluated, providing a novel perspective on the molecular mechanisms underlying SFTS.

## MATERIALS AND METHODS

### Patient and clinical sample information

According to the diagnostic criteria outlined in the Severe Fever with Thrombocytopenia Syndrome Guidelines (2010 edition), a total of 82 patients diagnosed with SFTS and 49 healthy controls were enrolled from Nanjing Drum Tower Hospital, Nanjing, China. The sample size was designed based on the primary endpoint of this study (evaluating the diagnostic efficacy of the biomarker) and the estimated effect value. The aim was to ensure that, at an *α* level of 0.05 (two-tailed) and with a statistical power of ≥80%, clinically significant differences between groups could be detected. All SFTS cases were confirmed by positive nucleic acid testing for SFTSV. Disease severity was classified at admission according to the 2010 guideline and adjudicated using documented clinical manifestations. Mild cases were defined by a body temperature below 38.0°C, accompanied by symptoms such as mild fatigue, general malaise, and gastrointestinal discomfort. Laboratory examinations revealed only mild reductions in white blood cell count and platelet count, typically with a platelet count above 100 × 10^9^/L. These patients typically exhibited clinical recovery within one week. Severe cases were characterized by high fever (typically 39–40°C or higher), profound fatigue, anorexia, dull facial expression, listlessness, skin ecchymosis, and neurological manifestations such as drowsiness, confusion, or increased muscle tone. Complications may include lung infection or bleeding in the digestive tract, lungs, or uterus. These patients were usually accompanied by at least two of the following laboratory abnormalities: significant thrombocytopenia (typically below 50 × 10^9^/L), markedly elevated transaminases (ALT/AST often exceeding five times the upper limit of normal), or coagulation dysfunction (e.g., prolonged APTT). In addition, severe cases may present with one or more of the following: coma, delirium, recurrent convulsions, shock, or multi-organ failure requiring intensive care unit monitoring and treatment. Fatal cases were defined as those who died during the course of SFTS.

### Collection and preparation of serum samples

Peripheral blood samples were collected from SFTS patients and allowed to clot at room temperature. After centrifugation at 1,710 × *g* for 3 min, the supernatant (serum) was carefully collected, aliquoted into RNase-free tubes, and stored at −80°C to prevent RNA degradation. To minimize potential bias, repeated freeze-thaw cycles were strictly avoided throughout sample handling. Samples were excluded if serum was unavailable or insufficient for RNA extraction, if key clinical data were missing, or if sample quality was inadequate for analysis.

### tsRNA sequencing and data analysis

A total of 20 serum samples were selected for tsRNA sequencing, comprising 5 healthy controls, 5 patients with mild disease, 5 with severe disease, and 5 who succumbed to the infection. This sample size was based on the design principles of the pilot study, aiming to efficiently screen out differentially expressed tsRNAs with the smallest sample set and provide candidate targets for subsequent large-sample validation, which is in line with the standard practice of such discovery studies. The criteria used for identifying differentially expressed tsRNAs were as follows: (i) fold change >2; (ii) *P* value < 0.05; (iii) no expression values equal to 0 in any group; and (iv) presence of differential expression across multiple comparison combinations whenever possible. Based on these stringent criteria, the top six most significantly dysregulated tsRNAs were selected for further validation in an independent cohort of clinical samples.

### qRT-PCR

Total RNA was isolated from serum samples of SFTS patients using a commercial nucleic acid extraction kit, following the manufacturer’s instructions. First-strand cDNA synthesis was carried out using the miRNA 1st Strand cDNA Synthesis Kit (MR101-02, Vazyme), employing stem-loop reverse transcription primers under the following thermal cycling conditions: 25°C for 5 min, 50°C for 15 min, and 85°C for 5 min. qRT-PCR was performed on a Bio-Rad PCR system using ChamQ Universal SYBR qPCR Master Mix (Q711-02, Vazyme) with the optimized thermal profile: initial denaturation at 95°C for 30 s, followed by 40 cycles of 95°C for 5 s and 60°C for 30 s, and a final melt curve analysis from 65°C to 95°C in 0.5°C increments (5 s per step).

Cycle threshold (Ct) values were recorded, and any data with a coefficient of variation (CV) >7.5% across technical replicates were excluded from analysis. All primer sequences (detailed in Table 1) were synthesized by Sangon Biotech (Shanghai, China). For absolute quantification, standard curves were constructed using serially diluted synthetic tsRNA reference standards, which were subjected to the same reverse transcription and qPCR protocols, allowing for precise calculation of tsRNA concentrations in serum samples.

**TABLE 1:** Six candidate tsRNA sequences

No.	Name	tsRNA sequence	Reverse transcription primer	Forward primer	Reverse primer
1	tsRNA-5007a	GCGCCGCTGGTGTAG	GTCGTATCCAGTGCAGGGTCCGAGGTATTCGCACTGGATACGACCTACAC	CAACTATGCGCCGCTGGTGT	ATCCAGTGCAGGGTCCGAGG
2	tsRNA-5013c	CTAGGGGTATGATTCTCGC	GTCGTATCCAGTGCAGGGTCCGAGGTATTCGCACTGGATACGACGCGAGA	GCGCGCTAGGGGTATGAT	
3	tsRNA-5017b	GGGGGTGTAGCTCAGTGGT	GTCGTATCCAGTGCAGGGTCCGAGGTATTCGCACTGGATACGACACCACT	CTCGTGGGGGTGTAGCTC	
4	tsRNA-5027b	GTTTCCGTAGTGTAGTGGTTATC	GTCGTATCCAGTGCAGGGTCCGAGGTATTCGCACTGGATACGACGATAAC	AGCGCCTGTTTCCGTAGTGTAG	
5	tsRNA-5030c	TCCCTGGTGGTCTAGTGGTTAGGATTCG	GTCGTATCCAGTGCAGGGTCCGAGGTATTCGCACTGGATACGACCGAATC	AAGAGCGTTCCCTGGTGGTCT	
6	tsRNA-3002a	CCCGGACGAGCCCCC	GTCGTATCCAGTGCAGGGTCCGAGGTATTCGCACTGGATACGACGGGGGC	TGGCGATCCCGGACGA	

### Receiver operating characteristic (ROC) curve analysis

All statistical analyses were conducted using R software (version 4.5.1) (14). To evaluate the predictive performance of candidate biomarkers—including tsRNA-5017b, platelet count (PLT), aspartate aminotransferase (AST), and their combination, we constructed a multivariate logistic regression model. The predicted probabilities derived from this model were used to generate receiver operating characteristic (ROC) curves for assessing diagnostic performance.

The area under the ROC curve (AUC), sensitivity, and specificity were calculated for each individual biomarker and the combined model. The optimal cutoff value for each indicator was determined by maximizing the Youden index. Comparative analysis of ROC curves was performed to determine whether the multi-indicator panel offered superior predictive value compared to any single marker.

### Target prediction and gene enrichment analysis

Putative mRNA targets of differentially expressed tsRNAs were predicted using two widely recognized algorithms: miRanda and RNAhybrid (https://bibiserv.cebitec.uni-bielefeld.de/). The screening thresholds were set as follows: for miRanda, a binding score ≥150 and minimum free energy <−20 kcal/mol; for RNAhybrid, minimum free energy <−25 kcal/mol. Only targets identified by both algorithms were retained as high-confidence candidates. Notably, the miRanda score reflects the binding affinity between tsRNAs and their predicted mRNA targets, with higher scores indicating greater prediction confidence (15). Gene Ontology (GO) and Kyoto Encyclopedia of Genes and Genomes (KEGG) enrichment analyses were subsequently performed using the clusterProfiler package (version 4.9.3.002) in R. This tool enables statistical interpretation and visualization of functional profiles across gene sets or clusters, thereby facilitating the identification of biological processes, cellular components, and molecular pathways potentially regulated by the candidate tsRNAs (16).

### Statistical analysis

Statistical analyses were performed using GraphPad Prism 9. Intergroup comparisons were analyzed using the Kruskal-Wallis test. Spearman’s rank correlation was applied to assess correlations between tsRNA levels and clinical indicators. A *P* value < 0.05 was considered statistically significant. *P < 0.05, **P < 0.01, ***P < 0.001, and ****P < 0.0001.

## RESULTS

### Characteristics of tsRNA in the serum of SFTS patients

In this study, tsRNA sequencing was performed on serum samples from 15 patients with SFTS diagnosed at Nanjing Drum Tower Hospital between July and October 2023, along with 5 healthy controls. The average age of SFTS patients included in the sequencing cohort was 64.3 years (range: 51–85 years), with 5 males (33.3%) and 10 females (66.7%).

For subsequent clinical validation, an expanded cohort comprising 82 SFTS patients and 49 healthy controls was enrolled. The demographic and clinical characteristics of all participants are summarized in Table 2. Among the 82 patients with SFTS, the median age was 69.5 years (interquartile range: 57.0–76.0), with a gender distribution of 41 males (50%) and 41 females (50%). Based on clinical classification, 33 patients were classified as mild cases, 34 as severe cases, and 15 patients died during hospitalization. After classifying by disease severity, the clinical and demographic characteristics of all participants are summarized in Table S1.

**TABLE 2:** Clinical characteristics of the enrolled samples

Index	Sequencing cohort		*P* value	Verification cohort		*P* value
	SFTS	HC		SFTS	HC	
Cases	15	5	N/A^*a*^	82	49	N/A^*a*^
Gender (male/female)	5/15	2/3	0.833	41/41	24/25	0.575
Age (years)	64.27 ± 8.96	61.20 ± 2.77	0.468	69.50 (57.00, 76.00)	64.73 ± 6.49	0.059
Ct value	23.74 ± 4.72	>40	0.001	22.41 ± 6.56	>40	<0.001
WBC (10^9^/L)	2.80 (2.20, 4.45)	4.41 ± 0.35	0.221	2.90 (2.00, 4.25)	5.80 (5.10, 6.70)	<0.001
PLT (10^9^/L)	52.20 ± 20.08	237.80 ± 30.08	0.001	61.00 (35.00, 86.00)	221.08 ± 46.50	<0.001
CRP (g/L)	6.14 (3.25, 17.28)	0.01 (0.01, 0.01)	0.001	8.64 (3.80, 17.77)	0.10 (0.10, 0.60)	<0.001
ALT	106.00 (70.45, 143.50)	14.44 ± 1.33	0.001	65.95 (40.20, 121.25)	18.00 (14.20, 23.90)	<0.001
AST	323.00 (143.05, 526.60)	19.86 ± 2.11	0.005	196.65 (80.50, 450.75)	21.40 (18.90, 23.50)	<0.001
γ-GGT	51.00 (35.40, 178.55)	23.78 ± 7.39	0.007	40.00 (25.25, 68.10)	21.90 (15.60, 26.90)	<0.001
ALP	82.60 (62.50, 172.10)	76.16 ± 16.60	0.600	68.00 (58.10, 89.75)	73.10 ± 15.33	0.713
LDH	1,159.87 ± 752.79	148.80 ± 7.40	0.009	681.50 (449.00, 1,768.00)	183.65 ± 23.01	<0.001
PCT	0.27 (0.20, 0.38)	0.10 (0.10, 0.10)	0.037	0.29 (0.11, 0.43)	0.03 (0.02, 0.05)	<0.001
IL-6	55.45 (43.38, 89.72)	0.26 ± 0.05	<0.001	59.70 (19.37, 177.47)	0.21 ± 0.06	<0.001
PT	12.25 ± 1.56	12.00 ± 0.79	0.734	11.83 ± 0.89	11.60 ± 0.99	0.190
APTT	46.50 (31.35, 53.95)	23.30 ± 0.47	<0.001	42.15 (34.65, 48.40)	23.85 ± 1.01	<0.001
TT	27.50 (20.10, 35.60)	13.78 ± 0.31	0.001	26.75 (20.93, 38.98)	14.15 ± 0.63	<0.001
BNP	214.00 (35.35, 313.00)	5.80 ± 0.23	0.001	73.55 (21.90, 152.00)	6.00 (5.00, 9.00)	<0.001
CK	612.00 (299.00, 1,255.00)	22.20 ± 1.92	0.001	707.00 (376.50, 1,564.00)	29.00 (24.00, 96.00)	<0.001
CK-MB	23.14 ± 14.74	0.58 ± 0.23	0.002	28.00 (16.75, 53.75)	1.58 (0.92, 5.80)	<0.001
cTnT	0.06 (0.03, 0.15)	0.11 ± 0.11	0.768	0.03 (0.02, 0.06)	0.02 ± 0.01	0.414

^*a*^ N/A, not applicable.

**Fig 1:**
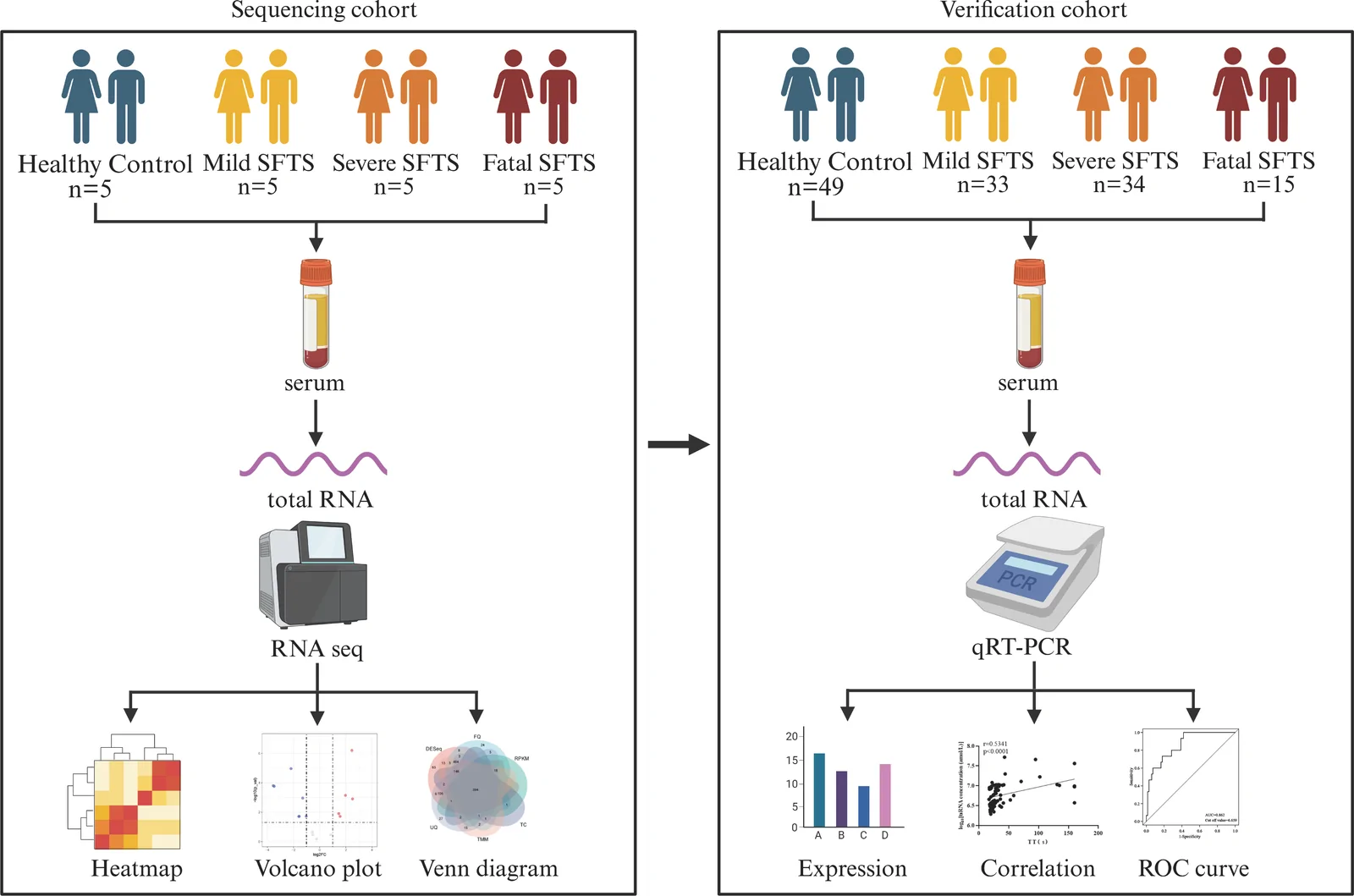
Discovery cohort Validation cohort. This sequencing cohort included five healthy controls, five patients with mild SFTS, five patients with severe SFTS, and five patients who died from SFTS. Total RNA was extracted from serum samples for RNA sequencing, followed by data analysis, including heat maps, volcano plots, and Venn diagrams. The validation cohort consisted of 49 healthy controls and 82 SFTS patients. Total RNA was extracted from serum samples for qRT-PCR analysis. Gene expression levels were measured, and correlation and ROC curves were generated to further validate. The 82 SFTS patients in the validation cohort included a subset from the preliminary validation cohort, ensuring comprehensive validation at different stages of SFTS.

To investigate the potential of serum tsRNA signatures as novel biomarkers for SFTS, we first conducted high-throughput small RNA sequencing on serum samples from 15 patients with SFTS and 5 healthy controls (HCs). Total RNA was extracted from serum, followed by next-generation sequencing to identify differentially expressed tsRNAs. Six candidate tsRNAs were subsequently selected for clinical validation by qRT-PCR (Fig. 1).

**Fig 2:**
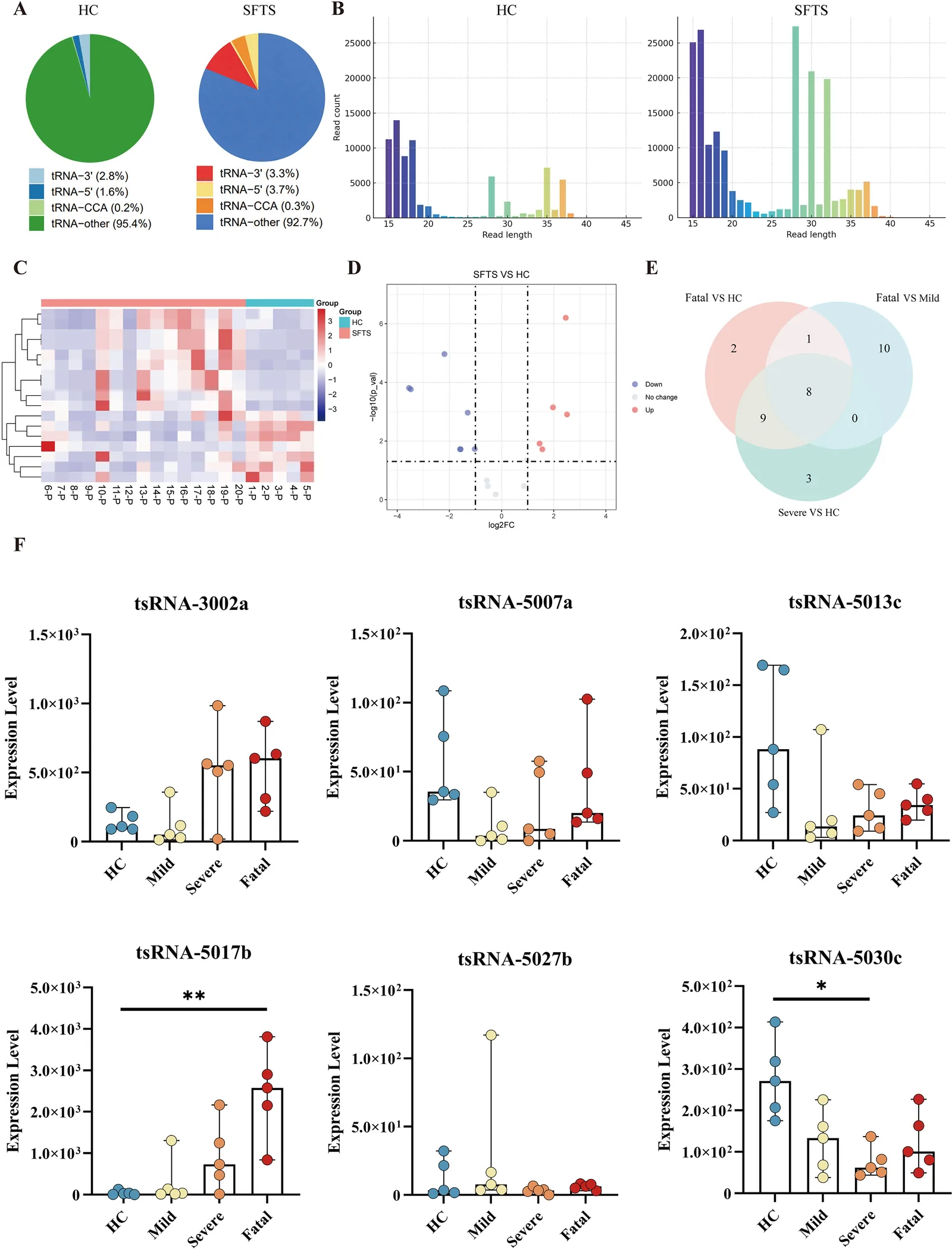
Differentially expressed tsRNAs in the serum of SFTS and HC individuals. (**A**) The species distribution characteristics of tsRNA in HC and SFTS patients. (**B**) The length distribution of tsRNA in HC and SFTS patients. (**C**) Heatmap of tsRNA differential expression between SFTS and HC. (**D**) Volcano plot of tsRNA differential expression between SFTS and HC. (**E**) Venn diagram of differential tsRNA in the fatal group and healthy group, fatal group and mild group, and severe group and healthy group. (**F**) Bar chart of the expression levels of tsRNA-3002a, tsRNA-5007a, tsRNA-5013c, tsRNA-5017b, tsRNA-5027b, and tsRNA-5030c in different types of SFTS. HC, healthy control; Mild, patients with mild SFTS; Severe, patients with severe SFTS; Fatal, patients who died from SFTS. Data are presented as the median. *P < 0.05 and **P < 0.01.

Based on their genomic origin within precursor or mature tRNA molecules, identified tsRNAs were categorized into four subtypes: 5′ tsRNA, 3′ tsRNA, 3′ tsRNA-CCA, and miscellaneous tsRNAs. Analysis of subtype distribution revealed a modest increase in the relative abundance of 5′ tsRNAs in SFTS patients compared to controls (Fig. 2A). Additionally, length distribution analysis indicated marked shifts between groups: tsRNA lengths in SFTS samples were predominantly enriched at 15–16 nucleotides and 28–32 nucleotides, with overall higher abundance than in HCs (Fig. 2B). Hierarchical clustering analysis demonstrated distinct global expression profiles, with samples forming clusters consistent with their clinical grouping (Fig. 2C). This observation suggests that tsRNA expression patterns reflect the host’s disease state and may serve as reliable molecular indicators of SFTS severity.

Differential expression analysis using the thresholds |fold change| > 2 and *P* < 0.05 identified 12 significantly dysregulated tsRNAs, of which 5 were upregulated and 7 were downregulated in SFTS patients relative to healthy controls (Fig. 2D). To further delineate the association between tsRNAs and disease progression, patients were stratified into mild, severe, and fatal subgroups. Venn diagram analysis revealed both unique and overlapping tsRNAs across these comparisons, reflecting disease-stage-specific expression shifts (Fig. 2E).

From the intersecting tsRNAs, six highly dysregulated tsRNAs—tsRNA-3002a, tsRNA-5007a, tsRNA-5013c, tsRNA-5017b, tsRNA-5027b, and tsRNA-5030c—were prioritized as top candidates for downstream validation based on their statistical significance and consistent differential expression across multiple subgroup comparisons. Their normalized expression profiles are illustrated in Fig. 2F.

### tsRNA-5017b was significantly elevated in serum samples from SFTS patients

**Fig 3:**
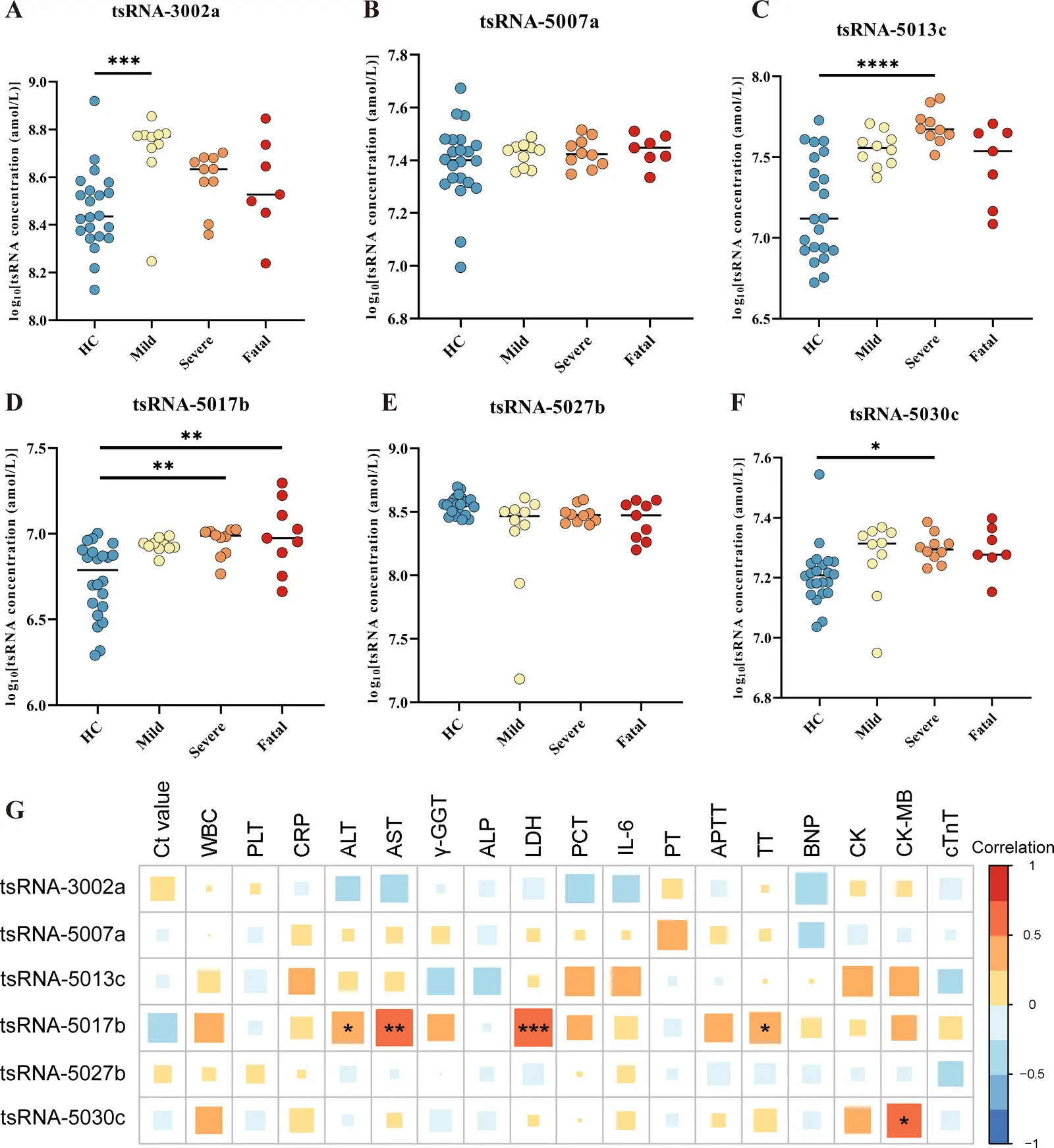
Clinical sample verification of the expression levels of differential tsRNAs. (**A–F**) Using qRT-PCR, we analyzed the levels of tsRNAs in the sera of 29 SFTS patients and 22 healthy controls. Intergroup comparisons were analyzed using the Kruskal-Wallis test. Data are presented as the median. (**G**) Heatmap of the correlations between six tsRNAs and clinical detection indicators. *P < 0.05, **P < 0.01, ***P < 0.001, and ****P < 0.0001.

To validate the findings from sequencing, we analyzed an independent cohort comprising 29 patients with SFTS and 22 healthy controls. qRT-PCR was used to assess serum levels of the six candidate tsRNAs. The results revealed that tsRNA-3002a, tsRNA-5013c, tsRNA-5017b, and tsRNA-5030c were elevated to varying degrees in SFTS patients compared to controls (Fig. 3A through F). Among these, tsRNA-5017b exhibited a stepwise increase in expression that paralleled disease severity, consistent with its trend observed in the initial sequencing data.

To further explore the clinical significance of tsRNA-5017b, we performed Spearman correlation analyses between its expression levels and key clinical parameters associated with disease severity. Notably, tsRNA-5017b expression correlated positively with alanine aminotransferase (ALT), aspartate aminotransferase (AST), lactate dehydrogenase (LDH), and thrombin time (TT) (Fig. 3G), indicating a potential association with hepatic injury and coagulation dysfunction in SFTS patients. These findings suggest that tsRNA-5017b may not only reflect disease presence but also mirror the extent of organ involvement, thus serving as a promising non-invasive biomarker for early risk stratification.

### tsRNA-5017b serves as a potential biomarker for SFTS disease severity

**Fig 4:**
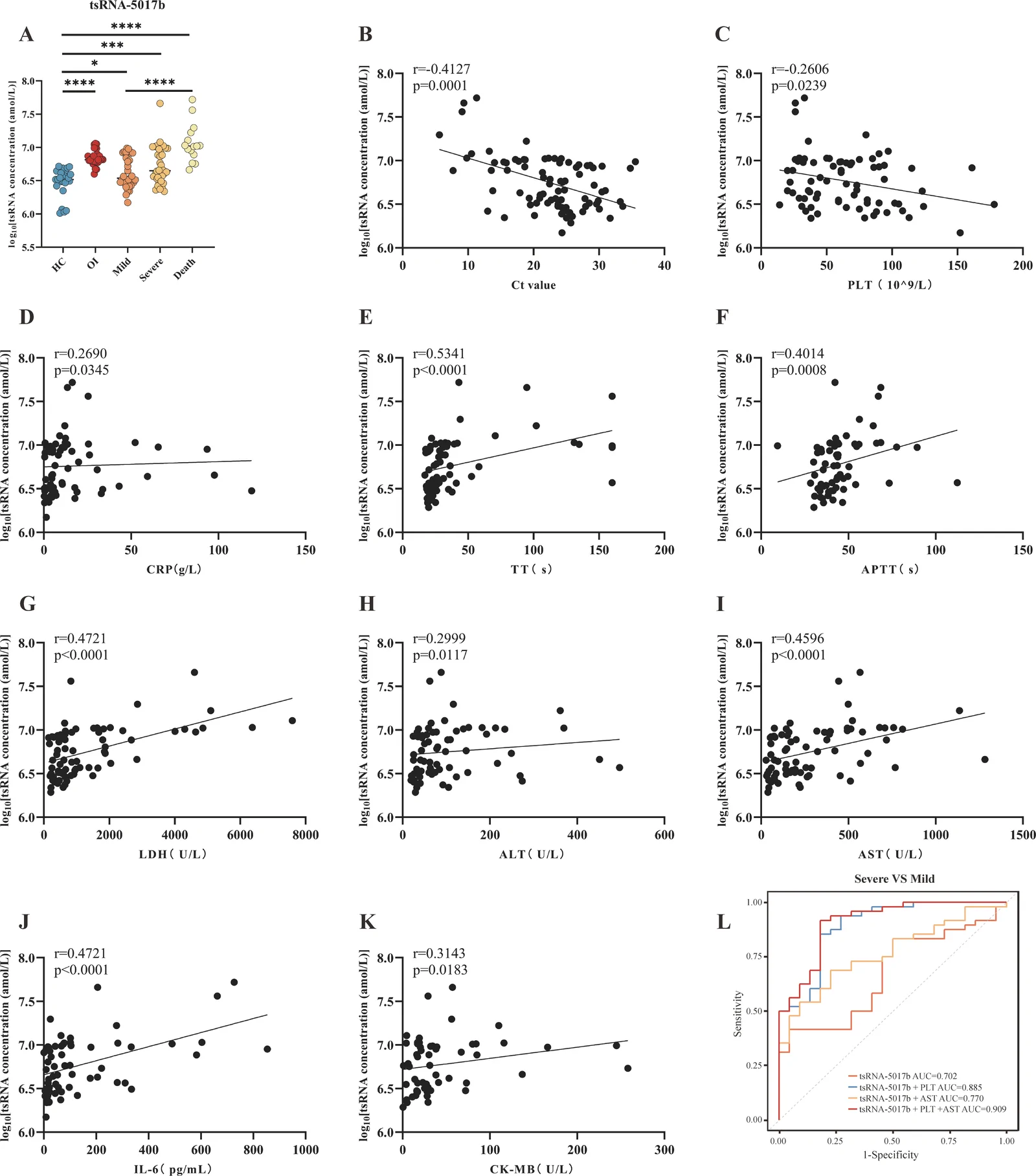
The application of serum tsRNA-5017b in predicting the severity of SFTS disease. (**A**) The concentration level of tsRNA-5017b increased in different types of SFTS. OI: Other viral infection groups. (**B–K**) Using the Spearman rank correlation test, the correlation between the laboratory indicators for assessing the severity of SFTS patients and the level of tsRNA-5017b was determined. Each chart indicates the *P* value and *r* value. (**L**) The ROC curve for the early prediction of the severity of SFTS patients’ disease by combining tsRNA-5017b with laboratory indicators. Data are presented as the median. *P < 0.05, ***P < 0.001, and ****P < 0.0001.

Building upon the initial sequencing results and preliminary clinical validation, we further explored the predictive potential of tsRNA-5017b in a larger cohort. A total of 82 SFTS patients and 27 healthy controls and 29 individuals with other viral infections—including HBV, HCV, Epstein-Barr virus (EBV), and cytomegalovirus (CMV)—were enrolled for qRT-PCR validation. The 27 healthy controls in the verification cohort are independent of the initial validation cohort, and the 82 SFTS patients include 29 SFTS patients from the initial validation cohort. qRT-PCR analysis revealed that tsRNA-5017b levels in the other infection (OI) group were significantly higher than those in healthy controls, indicating that tsRNA-5017b is generally upregulated following viral infection. Consistently, serum tsRNA-5017b levels in SFTS patients were markedly elevated compared with healthy controls and exhibited a progressive increase across disease severity categories, with the highest levels observed in the fatal group (Fig. 4A). Collectively, these findings demonstrate that, although tsRNA-5017b can be induced by multiple viral infections, its expression is particularly enriched in SFTS and positively correlates with clinical severity, supporting its potential utility as a biomarker for severity assessment at admission.

To investigate clinical relevance, Spearman correlation analysis was performed between tsRNA-5017b levels and key laboratory indicators. tsRNA-5017b was negatively correlated with platelet count and viral load (Fig. 4B and C). Conversely, tsRNA-5017b showed strong positive correlations with coagulation markers (TT and APTT), liver enzymes (ALT, AST, and LDH), inflammatory markers (CRP and IL-6), and creatine kinase-MB (CK-MB) (Fig. 4D through K), implicating its association with hepatic injury, coagulation dysfunction, inflammation, and potentially myocardial damage, further supporting its role as a marker of disease burden.

To assess its diagnostic performance, a receiver operating characteristic (ROC) curve was constructed. As a standalone biomarker, tsRNA-5017b yielded an AUC of 0.702, with a cutoff value of 6.949, sensitivity of 42.9%, and specificity of 93.9% (Fig. 4L). This indicates that tsRNA-5017b exhibits high specificity in identifying severe SFTS cases, although sensitivity remains limited when used alone.

Given that traditional clinical markers such as platelet count (PLT) and AST have previously been associated with SFTS severity (9, 17), we next evaluated whether combining these with tsRNA-5017b could enhance predictive performance. A multi-marker logistic regression model was developed. The AUC of the combined model with tsRNA-5017b + PLT reached 0.885 (sensitivity: 93.9%, specificity: 74.1%), and tsRNA-5017b + AST yielded an AUC of 0.770 (sensitivity: 66.7%, specificity: 81.5%). Notably, the triple-marker model integrating tsRNA-5017b, PLT, and AST achieved the best predictive performance, with an AUC of 0.909, sensitivity of 91.7%, and specificity of 81.8%.

These findings demonstrate that tsRNA-5017b, particularly in combination with routine clinical indicators, provides a robust and non-invasive tool for severity-associated risk stratification in SFTS. This integrated approach offers superior predictive power compared to individual biomarkers, supporting the clinical translational potential of tsRNA-based diagnostics in infectious diseases.

### Functional prediction and bioinformatic profiling of tsRNA-5017b

**Fig 5:**
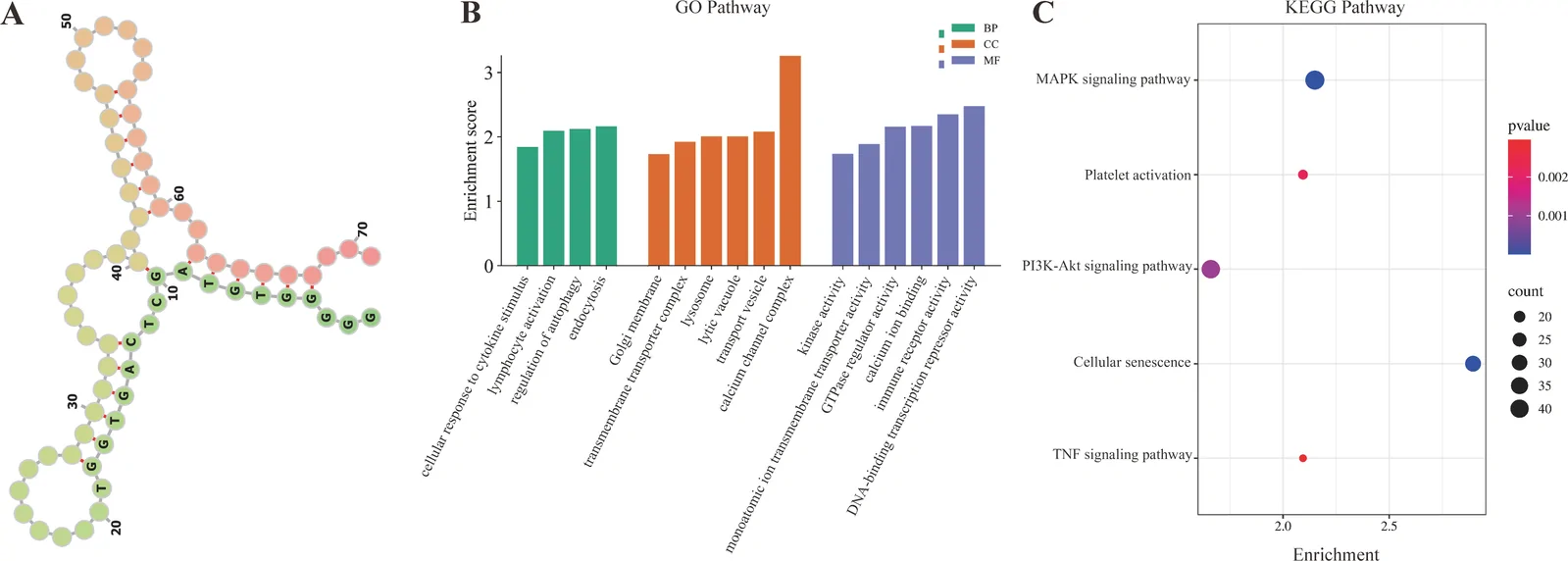
The biological function of tsRNA-5017b. (**A**) Secondary structure prediction of tsRNA-5017b. (**B**) GO functional enrichment analysis of potential target genes of tsRNA-5017b. (**C**) KEGG pathway analysis of potential target genes of tsRNA-5017b. GO, Gene Ontology; KEGG, Kyoto Encyclopedia of Genes and Genomes.

To investigate the structural and functional properties of tsRNA-5017b, we utilized the ViennaRNA Web Services to predict its secondary structure. The results indicated that tsRNA-5017b originates from a mature tRNA and belongs to the tRF-5 fragment class (Fig. 5A), suggesting it is derived from the 5′ end of the parent tRNA molecule. This classification implies potential regulatory functions beyond canonical tRNA roles, in line with emerging studies on tRNA-derived small RNAs.

To elucidate the potential biological roles of tsRNA-5017b, bioinformatic analyses were performed on its predicted target genes. Candidate mRNAs were identified using dual-algorithm prediction (miRanda and RNAhybrid), followed by Gene Ontology (GO) and Kyoto Encyclopedia of Genes and Genomes (KEGG) enrichment analyses. GO enrichment results revealed that the target genes were significantly associated with lymphocyte activation, autophagy regulation, lysosomal function, kinase activity, and calcium ion binding (Fig. 5B), implicating tsRNA-5017b in key immune and intracellular signaling processes.

Furthermore, KEGG pathway analysis highlighted strong enrichment in pathways involved in MAPK signaling, platelet activation, and PI3K-Akt signaling (Fig. 5C). These pathways are closely related to immune modulation, inflammation, and apoptosis—all of which are central to the pathogenesis of SFTS. Taken together, these findings suggest that tsRNA-5017b may exert pleiotropic effects through post-transcriptional regulation of genes involved in immune activation, cell survival, and inflammatory responses, providing a plausible mechanistic basis for its association with disease severity.

## DISCUSSION

SFTS is characterized by severe clinical outcomes such as hemorrhage, encephalitis, and multi-organ dysfunction, with mortality rates ranging from 16.2% to 27.0% across East Asia (18). Therefore, accurate early risk identification is crucial for reducing the mortality rate. Although several biomarkers and prediction models have been proposed, their clinical utility remains limited. Models based on factors such as age, CD4^+^ T cell count, IL-6 (19), or eosinophil and basophil counts (20) are susceptible to physiological and environmental fluctuations (21, 22), thereby reducing their stability and predictive accuracy. A recent study has developed a multi-layer perceptron model (UNION-SFTS) using traditional laboratory indicators, which has overcome the limitations of linear regression and significantly improved risk stratification (23). Other studies have also shown that changes in APTT are related to bleeding and poor prognosis (24), as well as the relationship between elevated cardiac injury marker troponin I (TnI) and increased mortality risk (25). Nevertheless, these efforts still rely primarily on conventional laboratory or clinical indicators, which are not disease-specific and remain vulnerable to confounding influences. To date, the role of tsRNAs in SFTS has not been investigated, underscoring the importance of the present study, which evaluates tsRNA-5017b as a novel severity-associated biomarker with the potential to enhance early risk assessment and inform clinical management of SFTS.

Given the emerging relevance of tsRNAs in diverse diseases, understanding their biological roles is essential. tsRNAs are an evolutionarily conserved class of non-coding RNAs derived from precursor or mature tRNAs and participate in cell proliferation, immune regulation, viral replication, and stress responses (5). Owing to their stable expression in body fluids and high interspecies conservation, tsRNAs show great promise as molecular biomarkers. Recent studies highlight their diagnostic and prognostic value in diseases such as lung cancer, systemic lupus erythematosus, and hematologic malignancies (26–29). Compared with traditional biomarkers, tsRNAs offer high stability, minimal invasiveness, and disease-specific expression. In infectious diseases, tsRNAs act as key regulators of immune responses, viral replication, and inflammation. For example, 5′ tsRNA-GluCTC promotes RSV replication by modulating host immune gene expression (30), and SARS-CoV-2 infection elevates tsRNA levels in nasopharyngeal swabs (8).

In our study, we report for the first time the detection of tsRNAs in the serum of patients with SFTS. Compared to healthy controls, multiple tsRNAs exhibited significantly altered expression levels in SFTS patients, including tsRNA-3002a, tsRNA-5007a, tsRNA-5013c, tsRNA-5017b, tsRNA-5027b, and tsRNA-5030c. Among these, tsRNA-5017b showed the most prominent upregulation. Notably, tsRNA-5017b levels were also elevated in patients with other viral infections, albeit to a lesser extent than in SFTS patients. This suggests a general activation of tsRNA expression upon viral challenge, yet the distinctly higher expression in severe and fatal SFTS cases highlights a disease-specific regulatory role, possibly linked to host immune dysregulation and viral replication dynamics. Of note, the expression of tsRNA-5017b was negatively correlated with viral load and PLT, and positively correlated with liver function markers (ALT, AST, and LDH), coagulation indicators (APTT and TT), the inflammatory cytokine IL-6, and the myocardial injury marker CK-MB, suggesting a strong association with disease severity.

Building on these findings, a predictive model integrating tsRNA-5017b with PLT and AST achieved the highest accuracy for assessing SFTS severity, with a sensitivity of 91.7% and a specificity of 81.8%. This three-indicator model outperformed the use of PLT and AST alone or the combination of tsRNA-5017b with any single indicator, demonstrating that incorporating tsRNA-5017b may improve early severity assessment. These observations align with previous reports (17) that established the prognostic significance of PLT and AST, further underscoring their key role as core indicators when integrated with tsRNA-5017b for accurate severity assessment. It is worth noting that, as an independent predictive indicator, tsRNA-5017b exhibits high specificity (93.9%) but moderate sensitivity (42.9%). This indicates that when used alone for screening or ruling out severe cases, tsRNA-5017b may not be sufficient to identify all high-risk patients, and there is a certain risk of missed diagnoses. However, its high specificity means that when the level of this marker is significantly elevated, the probability of the patient suffering from severe SFTS is very high. This characteristic makes it more suitable to be used as a supplement or combined indicator to the existing clinical risk assessment tools rather than completely replacing them. In this study, when tsRNA-5017b was combined with the conventional indicators PLT and AST, the sensitivity of the model significantly increased to 91.7%, while maintaining good specificity (81.8%). This combination strategy effectively compensates for the limitations of a single marker, forming a more robust predictive model, providing a more solid foundation for its potential clinical application.

Target gene enrichment analysis revealed that tsRNA-5017b is primarily involved in lymphocyte activation, regulation of autophagy, endocytosis, and the MAPK signaling pathway, suggesting that circulating tsRNAs may be closely associated with SFTSV infection, immune responses, and cytokine storm formation (9). A previous study has demonstrated that SFTSV induces lysosome-dependent autophagy and exploits the nonstructural protein NSs to evade innate antiviral immunity (31), and that infection triggers Ca^2+^ influx, which facilitates efficient viral replication (32). Consistent with these findings, tsRNA-5017b target genes are enriched in pathways related to Ca^2+^ binding and calcium channel complexes, implying a potential role in SFTSV replication. Moreover, in agreement with our results, SFTSV infection causes profound dysregulation of the host immune system, leading to significant disturbances in immune homeostasis and immune cell composition (33). Collectively, these observations indicate that tsRNA-5017b may contribute to the pathogenesis of SFTSV infection by participating in viral replication and modulating host immune responses. This provides a possible mechanism hypothesis for the observed clinical correlation. However, it must be clearly pointed out that the functional prediction results in the current study are mainly based on bioinformatics analysis and are still speculative. Future direct verification through *in vitro* and *in vivo* experiments is needed to clarify its specific mechanism of action.

This study is the first to report the potential value of tsRNAs as severity-associated biomarkers in SFTS, although their precise roles in disease progression remain to be fully elucidated. Future studies should aim to elucidate the mechanistic roles of tsRNAs in SFTSV replication, their interactions with host immunity, and their involvement in the cytokine storm. A deeper understanding of these processes would not only shed light on SFTSV pathogenesis but also facilitate the development of novel therapeutic strategies targeting tsRNA-mediated pathways. Nevertheless, our study has certain limitations. The sample size for clinical validation was relatively small, and larger cohorts are needed to confirm the clinical value and define a precise threshold of tsRNA-5017b for assessing disease severity.

In conclusion, this study is the first to identify tsRNAs in the serum of patients infected with SFTSV. Notably, the expression level of tsRNA-5017b was found to correlate strongly with disease severity. The model incorporating tsRNA-5017b demonstrated high sensitivity and showed robust discrimination for the early identification of severe cases. These findings suggest that tsRNAs hold significant promise as a novel molecular biomarker for severity assessment in SFTS.

## Data Availability

Data are available from the corresponding author upon reasonable request.

## SUPPLEMENTAL MATERIAL

Table S1 spectrum.00033-26-s0001.docx Comprehensive data set including demographic characteristics, experimental measurements, and statistical parameters (mean, standard deviation, *P*-value) for all study groups, supporting the main findings of the manuscript.

ASM does not own the copyrights to Supplemental Material that may be linked to, or accessed through, an article. The authors have granted ASM a non-exclusive, world-wide license to publish the Supplemental Material files. Please contact the corresponding author directly for reuse.
