# Supplementary material for: tRNA-derived small RNA, tsRNA-5017b, as a novel biomarker for predicting severity in severe fever with thrombocytopenia syndrome
Source: Microbiol Spectr. 2026 May 26;14(7):e00033-26. doi: 10.1128/spectrum.00033-26 (PMC13340336; doi:10.1128/spectrum.00033-26)
Supplement: Table S1 — Comprehensive data set including demographic characteristics, experimental measurements, and statistical parameters (mean, standard deviation, P-value) for all study groups, supporting the main findings of the manuscript. [file spectrum.00033-26-s0001.docx]

Table S1 . Clinical sample information of different types of SFTS.

| Index | Sequencing cohort | | | Verification cohort | | |
| --- | --- | --- | --- | --- | --- | --- |
|  | Mild | Severe | Fatal | Mild | Severe | Fatal |
| Cases | 5 | 5 | 5 | 33 | 34 | 15 |
| Gender （male/female） | 2/3 | 2/3 | 1/4 | 16/17 | 17/17 | 8/7 |
| Age（year） | 63.60 ± 5.98 | 62.20 ± 7.46 | 67.00 ± 13.21 | 63.30 ± 12.16 | 68.50 ± 8.96 | 74.00 (71.00, 79.00) |
| Ct value | 24.98 ± 4.63 | 23.22 (22.91, 25.96) | 20.52 ± 3.91 | 25.99 ± 4.30 | 21.10 ± 6.61 | 17.51 ± 6.61 |
| WBC（10^9/L） | 2.76 ± 0.94 | 6.42 ± 4.64 | 2.90 ± 1.16 | 2.90(2.05, 4.75) | 2.90 (1.90, 4.00) | 2.70 (1.95, 4.95) |
| PLT（10^9/L） | 70.40 ± 32.76 | 37.40 ± 12.66 | 30.40 ± 12.82 | 93.59 ± 34.84 | 38.00 (28.00, 60.00) | 52.67 ± 23.42 |
| CRP（g/L） | 7.37 (3.00, 8.87) | 3.49 (2.00, 4.10) | 9.87 (6.14, 24.77) | 4.68(2.85, 10.40) | 10.55 (3.93, 25.83) | 12.10 (9.69, 23.09) |
| ALT | 66.44 ± 40.04 | 123.70 ± 70.86 | 139.00 (106.00, 172.10) | 47.50(29.50, 77.00) | 71.95 (42.33, 135.00) | 112.50 (75.90, 174.25) |
| AST | 141.10 (138.00, 145.00) | 316.42 ± 225.11 | 422.20 (391.00, 631.00) | 85.00(56.00, 208.50) | 217.00 (140.00, 513.80) | 517.14 ± 348.64 |
| γ-GGT | 35.80 (35.00, 48.30) | 116.88 ± 93.72 | 145.60 ± 154.36 | 32.50(24.25, 46.25) | 34.25 (24.15, 77.00) | 51.65 (45.00, 91.35) |
| ALP | 79.00 (61.00, 90.80) | 106.36 ± 59.41 | 187.32 ± 138.61 | 68.42 ± 14.74 | 68.00 (57.50, 93.10) | 75.50 (59.30, 124.30) |
| LDH | 695.00 (543.00, 726.00) | 983.00 ± 492.05 | 1404.80 ± 401.17 | 445.00(260.00, 620.00) | 972.00 (609.00, 1874.00) | 1765.00 (909.25, 2854.75) |
| PCT | 0.32 (0.23, 3.58) | 0.14 ± 0.09 | 0.38 (0.35, 0.56) | 0.11(0.06, 0.23) | 0.35 (0.21, 0.78) | 0.41 (0.32, 8.52) |
| IL-6 | 39.39 ± 15.89 | 49.78 ± 5.00 | 377.36 (89.72, 702.40) | 16.40(10.79, 32.78) | 75.81 (41.26, 204.96) | 179.84 (79.51, 603.72) |
| PT | 11.64 ± 1.66 | 11.74 ± 1.10 | 13.38 ± 1.46 | 11.56 ± 0.83 | 11.93 ± 1.05 | 12.03 ± 0.46 |
| APTT | 33.16 ± 10.54 | 44.16 ± 16.60 | 72.48 ± 34.00 | 35.54 ± 4.88 | 43.45 (38.45, 53.98) | 45.20 (43.10, 60.25) |
| TT | 19.60 (19.60, 20.60) | 31.22 ± 11.06 | 33.50 (33.30, 35.40) | 20.10(19.80, 25.10) | 29.90 (23.05, 40.52) | 42.90 (33.50, 86.40) |
| BNP | 366.07 ± 312.06 | 19.35 (14.48, 71.55) | 608.00 ± 643.20 | 11.00(6.00, 41.35) | 94.60 (40.40, 173.00) | 144.00 (22.20, 215.00) |
| CK | 2265.67 ± 3573.05 | 416.00 ± 240.23 | 1233.33 ± 106.17 | 345.00(228.50, 555.75) | 1064.50 (452.75, 2483.25) | 748.00 (629.00, 839.50) |
| CK-MB | 21.43 ± 15.62 | 15.21 ± 6.76 | 36.00 ± 16.52 | 24.93 ± 15.97 | 32.00 (21.00, 76.50) | 18.00 (9.62, 35.75) |
| cTnT | 0.05 ± 0.03 | 0.07 ± 0.08 | 0.15 (0.09, 0.66) | 0.01 ± 0.01 | 0.03 (0.02, 0.05) | 0.06 (0.03, 0.11) |
